# Lipids, apolipoproteins, carbohydrates, and risk of hematological malignancies

**DOI:** 10.1007/s10654-025-01207-y

**Published:** 2025-03-04

**Authors:** Qianwei Liu, Dang Wei, Niklas Hammar, Yanping Yang, Maria Feychting, Zhe Zhang, Göran Walldius, Karin E. Smedby, Fang Fang

**Affiliations:** 1 Department of Hematology, Nanfang Hospital, Southern Medical University, No.1838, North Guangzhou Avenue, Guangzhou, Guangdong Province 510515 China; 2 Institute of Environmental Medicine, Karolinska Institutet, Stockholm, Sweden; 3 Department of Otolaryngology-Head & Neck Surgery, First Affiliated Hospital of Guangxi Medical University, Nanning, Guangxi China; 4 Clinical Epidemiology Division, Department of Medicine Solna, Karolinska Institutet, and Department of Hematology, Karolinska University Hospital, Stockholm, Sweden

**Keywords:** Hematological malignancy, Lipids, Apolipoproteins, Metabolism, Cohort study

## Abstract

Previous studies have investigated the role of metabolic factors in risk of hematological malignancies with contradicting findings. Existing studies are generally limited by potential concern of reverse causality and confounding by inflammation. Therefore, we aimed to investigate the associations of glucose, lipid, and apolipoprotein biomarkers with the risk of hematological malignancy. We performed a study of over 560,000 individuals of the Swedish AMORIS cohort, with measurements of biomarkers for carbohydrate, lipid, and apolipoprotein metabolism during 1985–1996 and follow-up until 2020. We conducted a prospective cohort study and used Cox models to investigate the association of nine different metabolic biomarkers (glucose, total cholesterol (TC), low-density lipoprotein cholesterol (LDL-C), high-density lipoprotein cholesterol (HDL-C), LDL-C/HDL-C, triglyceride (TG), apolipoprotein B (ApoB), apolipoprotein A-I (ApoA I), and ApoB/ApoA-I) with risk of hematological malignancy, after excluding the first five years of follow-up and adjustment for inflammatory biomarkers. We observed a decreased risk of hematological malignancy associated with one SD increase of TC (HR 0.93; 95% CI 0.91–0.96), LDL-C (HR 0.94; 95% CI 0.91–0.97), HDL-C (HR 0.92; 95% CI 0.86–0.99), and ApoA-I (HR 0.96; 95% CI 0.93–0.996). Our study highlights a decreased risk of hematological malignancy associated with a higher level of TC, LDL-C, HDL-C, and ApoA-I.

**Supplementary Information:**

The online version contains supplementary material available at 10.1007/s10654-025-01207-y.

## Introduction

Hematological malignancies are a group of biologically heterogenous disorders (e.g., acute myeloid leukemia, acute myeloid leukemia, multiple myeloma, Hodgkin lymphoma, non-Hodgkin lymphoma, and chronic lymphoid leukemia), characterized by dysfunction of the hematopoietic system [1]. Despite rapid development of treatment approaches, several hematological malignancy types remain a substantial public health concern and contributor to the global burden of disease [2]. Although previous studies have suggested potential contribution of lifestyle factors in the risk of some subtypes of hematological malignancies (e.g., obesity in non-Hodgkin lymphoma [3] and smoking in Hodgkin lymphoma [4]), large knowledge gaps remain regarding underlying etiology and risk factors of many hematological malignancies. Therefore, it is crucial to elucidate disease mechanisms, to improve prevention and patient survival as well as to reduce healthcare cost.

Experimental evidence has suggested an important role of metabolism alteration in the initiation and development of hematological malignancy [5–8]. Clinical and epidemiological studies have also proposed an association between lipid and carbohydrate metabolism and risk of different hematological malignancies, with, however, inconsistent results. A summary of existing studies on metabolic biomarkers and the risk of hematological malignancies is presented in Supplementary Table S1. Most existing studies have some methodological shortcomings, e.g., a relatively small sample size or a cross-sectional design [9–22]. Several prospective cohort studies have also investigated the association between metabolic biomarkers and risk of hematological malignancies [22–33]. However, these studies either did not consider lag time between measurement of biomarkers and diagnosis of hematological malignancy or used a relatively short lag time in the analysis [22–33], leaving reverse causation a potential concern. In addition, existing studies generally lacked careful control of important confounding factors, such as chronic inflammation [22–33], which might have distorted the observed association between metabolic biomarkers and hematological malignancy. Moreover, most existing cohort studies have mainly focused on one or a few specific metabolic biomarkers, leaving the interactive impact among different metabolic biomarkers largely unknown.

To this end, we performed a population-based cohort study in Sweden with more than 560,000 participants and a follow-up of more than 30 years to investigate the associations of carbohydrate, lipid, and apolipoprotein biomarkers with the subsequent risk of hematological malignancy. In this study, we hypothesized an inverse association of lipid and apolipoprotein biomarkers with risk of hematological malignancy, and no association between carbohydrate biomarkers and risk of hematological malignancy. We performed analyses to address concerns of reverse causality and potential confounding by inflammation, as well as to test potentially independent roles of different metabolic biomarkers after adjusting them for one another, on the risk of hematological malignancy.

## Methods

### Study design

The Swedish population-based AMORIS (Apolipoprotein-Related Mortality Risk) cohort was originally designed to investigate the role of blood metabolic and inflammatory biomarkers in the process of developing chronic disorders [34]. In the present study we used this cohort to investigate the associations between different metabolic biomarkers and the subsequent risk of hematological malignancy. The AMORIS cohort included 812,073 Swedish individuals predominantly residing in the Stockholm area who had undergone a health examination during the period of 1985–1996. The health examinations included blood collection and were conducted through occupational health check-ups or by outpatient hospital visits. All laboratory analyses were performed using consistently implemented and well-documented laboratory methods by one and the same clinical laboratory, the Central Automation Laboratory (CALAB), Stockholm, Sweden [34]. 

Using the unique Swedish personal identity numbers assigned to all Swedish residents, each participant was followed from first blood collection until December 31, 2020, through cross-linking the study cohort to the Swedish Cancer Register (for identification of incident hematological malignancy) and the Causes of Death and the Total Population Registers (for identification of death and migration status). The inclusion criteria of the study included (1) age at 20 years or above; (2) having at least one measurement of the studied biomarkers; and (3) free of cancer at the time of the baseline health examination (i.e., first blood collection). Individuals who did not meet the inclusion criteria were excluded (*N* = 248,885), leaving a total of 563,188 individuals in the final analysis.

Incident hematological malignancy was identified through the Swedish Cancer Register, using the Swedish 9th revision of the International Classification of Diseases (ICD) codes (ICD-9) as well as the 2nd ICD codes for oncology (ICD-O-2). The Swedish Cancer Register was established in 1958 and has included since then all new cases of cancer diagnosed in Sweden by mandatory registration. In this study, we analyzed any hematological malignancy as well as several common subtypes of hematological malignancy, namely acute myeloid leukemia, multiple myeloma, Hodgkin lymphoma, non-Hodgkin lymphoma, and chronic lymphoid leukemia. ICD codes for hematological malignancy and the studied subtypes are shown in Supplementary Table S2.

### Biomarkers of glucose, lipid and apolipoprotein metabolism

We collected information on date of blood collection and fasting status as well as serum levels of biomarkers. To ensure statistical power, we selected biomarkers that were available in more than 100,000 individuals. We obtained information of glucose (mmol/L) as a carbohydrate biomarker, total cholesterol (TC) (mg/dL), low-density lipoprotein cholesterol (LDL-C) (mg/dL), high-density lipoprotein cholesterol (HDL-C) (mg/dL), and triglyceride (TG) (mg/dL) as biomarkers for lipid metabolism, as well as apolipoprotein B (ApoB) (mg/dL) and apolipoprotein A-I (ApoA-I) (mg/dL) as biomarkers of apolipoprotein metabolism. LDL-C/HDL-C and ApoB/ApoA-I ratios were also calculated in the present study. We had information on serum levels of TC, TG and glucose for most of the study participants whereas the rest of biomarkers were available for only a smaller part of the participants [34]. HDL-C was directly measured among 15% of the participants and LDL-C was calculated using the Friedewald formula for these patients. For others, serum levels of LDL-C were calculated from TC, TG, and ApoA-I using the Jungner method, as previously validated [35, 36]. HDL-C was further calculated using the formula HDL-C = TC − 0.45 × TG − LDL-C.

### Covariates

In addition to sex, age, date and fasting status at blood collection, we also obtained information on country of birth and occupational status from the Swedish Censuses in 1970, 1980, 1985, and 1990 as well as from the Longitudinal Integration Database for Health Insurance and Social Market Studies (LISA) (since 1990) [37]. As previous research has suggested that inflammation might impact the level of cholesterol [38], we also identified health conditions related to chronic inflammation, including autoimmune disorders and infections, at the time of blood collection, from the Swedish Patient Register, which has since 1964 included information on inpatient care records (nationwide since 1987) and since 2001 outpatient specialist care (> 80% nationwide).^39^ ICD codes used to identify autoimmune disorders and infection are listed in Supplementary Table S3. Finally, we also collected data on several blood inflammatory biomarkers, including albumin (g/l), haptoglobin (g/l) and C-reaction protein (not high sensitive; mg/l), available in the AMORIS cohort [34]. 

### Statistical analysis

#### Prospective cohort study

We first described baseline characteristics of the cohort participants and then assessed the associations of glucose, lipids, and apolipoproteins with the subsequent risk of hematological malignancy. For each biomarker, the follow-up time of the study participants started from the time of first blood collection of the biomarker of interest (baseline), until the diagnostic date of hematological malignancy, emigration, death, or 31st December 2020, whichever came first. To alleviate the concern of reverse causation, only the first blood sample was used as the exposure, although participants of the AMORIS cohort could have more than one blood sample during the recruitment period. We disregarded the subsequent biomarker measurements in this analysis, assuming that measurements closer to the diagnosis of hematological malignancy are more likely to be secondary biological changes of the underlying malignancy. To further avoid potential reverse causality, we excluded the first five years of follow-up from this analysis.

We used Cox models to investigate the associations between the studied biomarkers and the subsequent risk of hematological malignancy by calculating hazard ratios (HRs) and their two-sided 95% confidence intervals (CIs). We first included each biomarker as a continuous variable in the Cox models to assess the association per standard deviation (SD) increase in the biomarker. To reduce potential bias due to continuous representation of the biomarkers, we also analysed each biomarker as a categorical variable by comparing the higher quartiles (Q2-Q4) with the lowest quartile (Q1). Attained age was used as the underlying time scale. In all analyses, we adjusted for sex (male or female), age and fasting status (overnight fasting versus non-fasting) at first blood collection, occupation, and country of birth. We first performed analysis for any hematological malignancy and then for acute myeloid leukemia, multiple myeloma, Hodgkin lymphoma, non-Hodgkin lymphoma, and chronic lymphoid leukemia, separately.

To evaluate the robustness of the findings, we performed a number of supplementary and sensitivity analyses. As abnormal levels of metabolic biomarkers might be caused by inflammation [38], which might also be related to the risk of hematological malignancy, we additionally adjusted for history of autoimmune disorders, history of infections, and blood levels of albumin, haptoglobin, and C-reaction protein. Information on these covariates was ascertained at the time of first blood collection or close to the first blood collection. To assess potential interaction among metabolic biomarkers, we conducted an analysis to evaluate potential correlations among studied biomarkers. We further investigated the association of one metabolic biomarker with hematological malignancy, by further adjusting for level of other metabolic biomarkers. As some of the health examinations were according to referrals from outpatient visits whereas majority were according to occupational health check-up, to evaluate potential indication bias, we performed an analysis restricting to first blood collection in relation to an occupational health check-up (i.e., due to screening reasons). To evaluate potential false positive findings due to multiple testing, we applied Bonferroni correction to adjust for level of statistical significance in a sensitivity analysis. Further, we used each biomarker as a categorical variable according to clinical cutoffs to explore risk of hematological malignancy in relation to clinically abnormal levels of the studied biomarkers. Finally, we conducted sensitivity analyses by further adjusting for diagnosis of diabetes (*N* = 563,188), BMI (*N* = 104,936), or smoking status (*N* = 38,648).

#### Nested case-control study

To investigate the temporal pattern of the studied biomarkers before the diagnosis of hematological malignancy, we performed a nested case-control study within the above cohort. We defined all cases of hematological malignancy ascertained during follow-up as cases, and the diagnostic date of hematological malignancy as the index date for these cases. For each biomarker, we used the incidence density sampling method to randomly select 25 controls (i.e., participants who were not censored and free of cancer on the index date of their matched cases) for each case and individually matched the controls to the case by sex, year of birth, and calendar year of first blood collection. We then analyzed all blood samples with a measurement of the specific biomarker during the 30 years before the index date for both cases and controls and used locally weighted scatterplot smoothing to visualize the mean levels of these biomarkers by time to index date among cases and controls, separately.

This study was approved by the Swedish Ethical Review Authority (DNR 2018/2401-31). All statistical analyses were conducted using SAS 9.4 (SAS Institute, Cary, NC) and Stata 16.1 (StataCorp, Texas, USA). The significance level was set at a two-sided *p* < 0.05 and Wald test was used to determine P values for all analyses.

## Results

Table 1 summarizes characteristics of the study participants at first blood collection. 53.7% of the participants were male and the mean age at first blood collection was 45.1 years. Most of the participants were born in Sweden and gainfully employed at the time of blood collection.

**Table 1:** Baseline characteristics of the cohort participants

Characteristics	Entire cohort *N* = 561,388 (100%)	Male *N* = 301,669 (53.7%)	Female *N* = 259,719 (46.3%)
**Age at first blood collection, mean (SD)**	45.1 (14.0)	44.4 (13.2)	45.8 (14.7)
Country of birth, N (%)			
Sweden	480,451 (85.6%)	262,281 (86.9%)	218,170 (84.0%)
Other Nordic countries	35,876 (6.4%)	16,025 (5.3%)	19,851 (7.6%)
Other or unknown	450,61 (8.0%)	23,363 (7.7%)	21,698 (8.4%)
**Occupational status**			
Employed	486,116 (86.6%)	270,750 (89.8%)	215,366 (82.9%)
Unemployed or unknown	75,272 (13.4%)	30,919 (10.2%)	44,353 (17.1%)
**Biomarkers of carbohydrate metabolism, mean (SD)**			
Glucose in mmol/l (*N* = 535,733)	4.99 (1.29)	5.11 (1.38)	4.85 (1.15)
Biomarkers of lipid metabolism, mean (SD)			
TC in mmol/l (*N* = 556,849)	5.59 (1.17)	5.60 (1.15)	5.57 (1.18)
LDL-C in mmol/l (*N* = 229,824)	3.69 (1.10)	3.75 (1.06)	3.61 (1.14)
HDL-C in mmol/l (*N* = 229,434)	1.52 (0.45)	1.38 (0.41)	1.70 (0.43)
LDL-C/HDL-C ratio^a^ (*N* = 229,140)	1.29 (0.73)	1.46 (0.73)	1.06 (0.66)
TG in mmol/l^a^ (*N* = 556,347)	0.15 (0.82)	0.33 (0.84)	-0.05 (0.74)
**Biomarkers of apolipoprotein metabolism, mean (SD)**			
ApoA-I in g/l (*N* = 202,661)	1.42 (0.24)	1.36 (0.21)	1.51 (0.24)
ApoB in g/l (*N* = 190,013)	1.25 (0.36)	1.30 (0.36)	1.20 (0.36)
ApoB/ApoA-I ratio^a^ (*N* = 180,096)	-0.22 (0.49)	-0.10 (0.46)	-0.37 (0.49)

^a^ Logarithmic transformation (log2) was used for the variables of LDL-C/HDL-C ratio, TG, and ApoB/ApoA-I ratio

TC: total cholesterol; LDL-C: low-density lipoprotein cholesterol; HDL-C: high-density lipoprotein cholesterol; TG: triglycerides; ApoA-I: apolipoprotein A-I; ApoB: apolipoprotein B

### Prospective cohort study

We observed a decreased risk of any hematological malignancy in relation to one SD increase of TC (HR 0.93; 95% CI 0.91–0.96), LDL-C (HR 0.94; 95% CI 0.91–0.97), HDL-C (HR 0.92; 95% CI 0.86–0.99), and ApoA-I (HR 0.96; 95% CI 0.93–0.996) (Table 2). When analyzing subtypes of hematological malignancy, we observed an inverse association of HDL-C with acute myeloid leukemia (HR 0.86; 95% CI 0.75–0.97), of TC (HR 0.89; 95% CI 0.84–0.94) and LDL-C (HR 0.90; 95% CI 0.83–0.98) with multiple myeloma, and of TC (HR 0.95; 95% CI 0.92–0.98), TG (HR 0.96; 95% CI 0.93–0.997) and ApoA-I (HR 0.93; 95% CI 0.88–0.99) with non-Hodgkin lymphoma. In the analysis comparing the upper three quartiles (Q2-4) to the bottom quartile (Q1) of the biomarkers (Table 3), a higher level of TC (HR 0.92; 95% CI 0.87–0.98), LDL-C (HR 0.86; 95% CI 0.79–0.93), HDL-C (HR 0.90; 95% CI 0.83–0.96), TG (HR 0.94; 95% CI 0.88–0.99), and ApoA-I (HR 0.88; 95% CI 0.82–0.95) was associated with a lower risk of any hematological malignancy.

**Table 2:** Incidence rates (IRs) per 100,000 person-years and hazard ratios (HRs) with 95% confidence intervals (CIs) of hematological malignancy per standard deviation increase of blood biomarkers of lipid, carbohydrate, and apolipoprotein metabolism

Biomarker (per standard deviation increase)	Any hematological malignancy			Acute myeloid leukemia		Multiple myeloma		Hodgkin’s lymphoma		Non-Hodgkin’s lymphoma		Chronic lymphoid leukemia	
	**N of cases**	**IR**	**HR (95% CI)** ^a^	**IR**	**HR (95% CI)** ^a^	**IR**	**HR (95% CI)** ^a^	**IR**	**HR (95% CI)** ^a^	**IR**	**HR (95% CI)** ^a^	**IR**	**HR (95% CI)** ^a^
Glucose	8,781	79.1	1.00 (0.98–1.02)	5.8	0.97 (0.88–1.06)	14.1	1.02 (0.97–1.07)	1.8	1.13 (1.01–1.25)	33.1	0.96 (0.93-1.00)	11.5	1.04 (0.99–1.10)
TC	9,112	79.6	**0.93 (0.91–0.96)**	5.8	0.92 (0.85-1.00)	14.3	**0.89 (0.84–0.94)**	1.8	0.97 (0.83–1.14)	33.3	**0.95 (0.92–0.98)**	11.5	0.98 (0.93–1.04)
LDL-C	3,942	88.8	**0.94 (0.91–0.97)**	6.3	0.91 (0.80–1.03)	16.1	**0.90 (0.83–0.98)**	2.0	0.95 (0.75–1.20)	37.0	0.96 (0.91–1.01)	13.2	1.00 (0.91–1.09)
HDL-C	3,931	88.6	**0.96 (0.92–0.99)**	6.3	**0.86 (0.75–0.97)**	16.1	0.96 (0.89–1.04)	2.0	1.10 (0.87–1.39)	36.9	0.95 (0.90-1.00)	13.2	0.96 (0.88–1.05)
LDL-C/HDL-C^b^	3,929	88.7	1.00 (0.96–1.03)	6.3	1.07 (0.99–1.16)	16.1	0.96 (0.88–1.04)	2.0	0.93 (0.74–1.18)	36.9	1.01 (0.96–1.06)	13.2	1.03 (0.95–1.12)
TG^b^	9,107	79.6	0.99 (0.96–1.01)	5.8	0.99 (0.91–1.08)	14.3	1.00 (0.95–1.05)	1.8	1.07 (0.92–1.24)	33.3	**0.96 (0.93–0.997)**	11.5	1.02 (0.96–1.08)
ApoA-I	3,534	89.3	**0.96 (0.93–0.996)**	6.7	0.88 (0.77–1.01)	16.1	0.96 (0.88–1.05)	2.0	1.06 (0.83–1.36)	37.4	**0.93 (0.88–0.99)**	13.0	1.01 (0.92–1.10)
ApoB	3,272	89.3	0.99 (0.95–1.02)	6.4	0.97 (0.84–1.11)	16.0	0.97 (0.89–1.06)	1.8	1.06 (0.82–1.38)	37.8	1.01 (0.95–1.07)	12.9	1.01 (0.92–1.11)
ApoB/ApoA-I^b^	3,122	90.2	0.98 (0.95–1.02)	6.6	1.02 (0.88–1.18)	16.1	1.00 (0.91–1.10)	1.9	1.13 (0.85–1.48)	37.7	1.04 (0.98–1.10)	13.3	0.99 (0.90–1.10)

^a^ Analyses were adjusted for sex, age at first blood collection, fasting status at first blood collection, occupational status, and country of birth

^b^ Logarithmic transformation (log2) was used to the variables of LDL-C/HDL-C ratio, TG, and ApoB/ApoA-I ratio

TC: total cholesterol; LDL-C: low-density lipoprotein cholesterol; HDL-C: high-density lipoprotein cholesterol; TG: triglycerides; ApoA-I: apolipoprotein A-I; ApoB: apolipoprotein B

Bold font indicates statistical significance

**Table 3:** Incidence rates (IRs) per 100,000 person-years and hazard ratios (HRs) with 95% confidence intervals (CIs) of hematological malignancy in relation to a higher (Q2-Q4) versus a lower (Q1) level of blood biomarkers of lipid, carbohydrate, and apolipoprotein metabolism

Biomarker	HR (95% CI)^a^
Glucose	
Q1 (ref)	1.0
Q2-Q4	0.95 (0.90-1.00)
TC	
Q1 (ref)	1.0
Q2-Q4	**0.92 (0.87–0.98)**
LDL-C	
Q1 (ref)	1.0
Q2-Q4	**0.86 (0.79–0.93)**
HDL-C	
Q1 (ref)	1.0
Q2-Q4	**0.90 (0.83–0.96)**
LDL-C/HDL-C^b^	
Q1 (ref)	1.0
Q2-Q4	0.93 (0.86–1.01)
TG^b^	
Q1 (ref)	1.0
Q2-Q4	**0.94 (0.88–0.99)**
ApoA-I	
Q1 (ref)	1.0
Q2-Q4	**0.88 (0.82–0.95)**
ApoB	
Q1 (ref)	1.0
Q2-Q4	0.92 (0.84–1.01)
ApoB/ApoA-I^b^	
Q1 (ref)	1.0
Q2-Q4	0.93 (0.85–1.02)

^a^ Analyses were adjusted for sex, age at first blood sampling, fasting status at first blood sampling, occupational status, and country of birth

^b^ Logarithmic transformation (log2) was used to the variables of LDL-C/HDL-C ratio, TG, and ApoB/ApoA-I ratio

TC: total cholesterol; LDL-C: low-density lipoprotein cholesterol; HDL-C: high-density lipoprotein cholesterol; TG: triglycerides; ApoA-I: apolipoprotein A-I; ApoB: apolipoprotein B

Bold font indicates statistical significance

Largely similar results were observed after additional adjustment for history of autoimmune disorders, history of infections, or inflammatory biomarkers (Table S4). The studied metabolic biomarkers are correlated with each another (Table S5); however, adjusting the metabolic biomarkers for one another in the analyses yielded similar results (Table S6). The results did not vary greatly after restricting the analyses to biomarkers measured in relation to an occupational health check-up (Table S7). Although we did not adjust for multiple testing, the sensitivity analysis with Bonferroni correction still noted an inverse association between TC and LDL-C and risk of any hematological malignancy (Table S8). In the analysis by clinical cutoffs of the biomarkers, individuals with a clinically abnormally high level of glucose (HR 0.92; 95% CI 0.85–0.997) had a lower risk of hematological malignancy, compared with individuals with normal range of glucose (Table S9). In addition, individuals with a clinically abnormally high level of TG had a higher risk of Hodgkin lymphoma (HR 1.63; 95% CI 1.03–2.58) and chronic lymphoid leukemia (HR 1.14; 95% CI 1.003–1.29). Estimates did not vary greatly after adjustment for diagnosis of diabetes, BMI, or smoking (Table S10).

### Nested case-control study

**Fig. 1:**
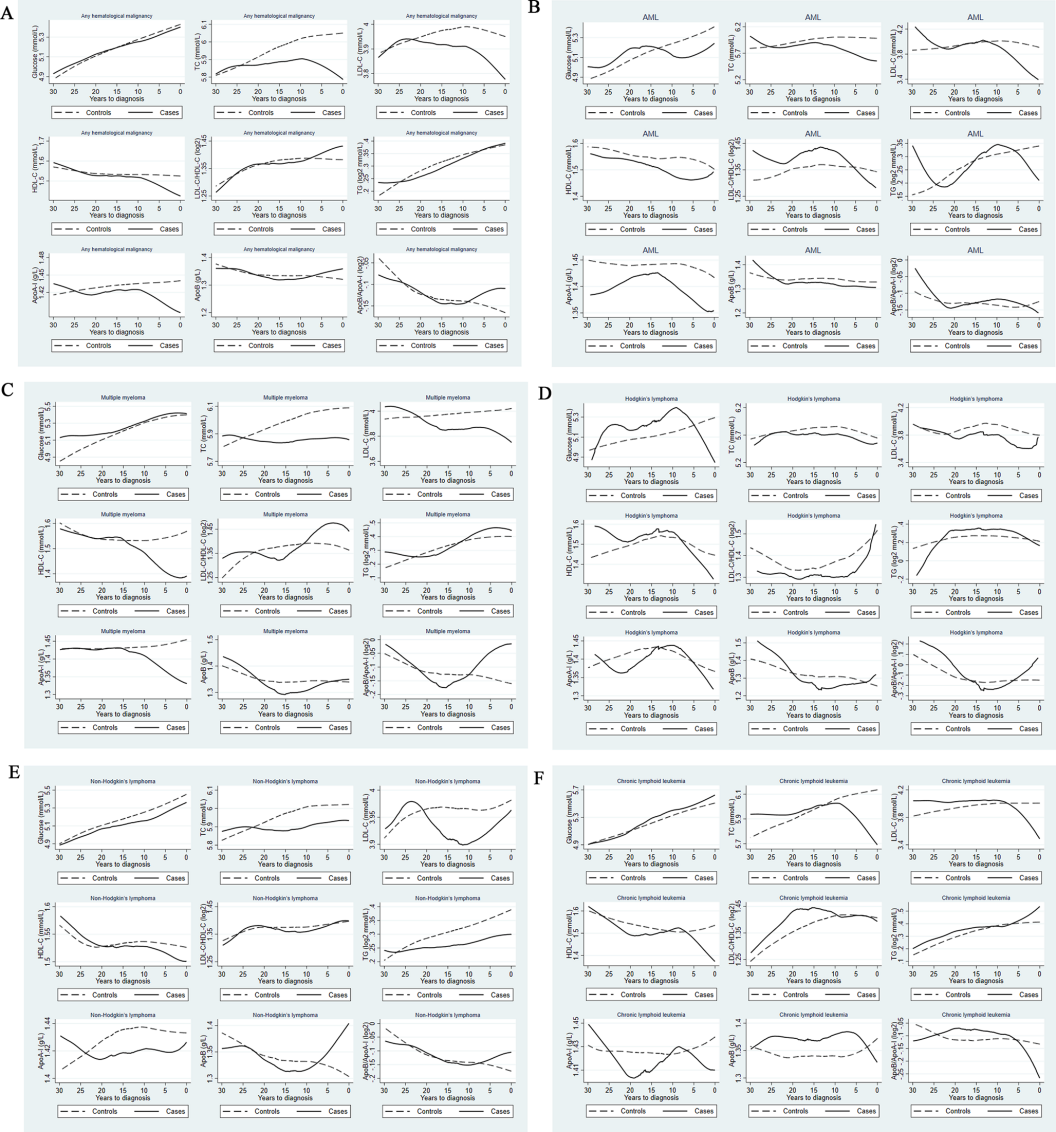
Mean concentrations of blood biomarkers of lipid, carbohydrate, and apolipoprotein metabolism during the 30 years before the diagnosis of hematological malignancy, comparing patients with hematological malignancy (solid line) to the matched controls (dashed line) **A**. Any hematological malignancy; **B**. Acute myeloid leukemia; **C**. Multiple myeloma; **D**. Hodgkin lymphoma; **E**. Non-Hodgkin lymphoma; **F**. Chronic lymphoid leukemia The curves were generated using the locally weighted scatterplot smoothing methods for the mean concentrations based on all available blood samples during the 30-year period, for both cases of hematological malignancy and their matched controls. TC: total cholesterol; LDL-C: low-density lipoprotein cholesterol; HDL-C: high-density lipoprotein cholesterol; TG: triglycerides; ApoA-I: apolipoprotein A-I; ApoB: apolipoprotein B

The mean levels of the metabolic biomarkers during 30 years before the index date of the cases and controls are shown in Fig. 1. We observed a lower level of TC, LDL-C, HDL-C, and ApoA-I more than 20 years before diagnosis among individuals with any hematological malignancy, compared to their matched controls (Fig. 1A). In subgroup analysis, we also found a persistently lower level of TC in patients with AML, multiple myeloma, Hodgkin lymphoma or non-Hodgkin lymphoma, a lower level of LDL-C in patients with multiple myeloma, Hodgkin lymphoma or non-Hodgkin lymphoma, a lower level of HDL-C in patients with AML, and a lower level of ApoA-I in patients with AML or non-Hodgkin lymphoma, more than 20 years before diagnosis (Fig. 1B-E). However, we observed no clear difference of the studied biomarkers between patients with chronic lymphoid leukemia and their matched controls (Fig. 1F).

## Discussion

In a large cohort with more than 30 years of follow-up, we observed a lower risk of hematological malignancy in relation to a higher level of TC, LDL-C, HDL-C, and ApoA-I. We also noted an inverse association of HDL-C with acute myeloid leukemia, of TC and LDL-C with multiple myeloma, and of TC, TG and ApoA-I with non-Hodgkin lymphoma. In addition, we observed persistently lower levels of these biomarkers more than 20 years before diagnosis among individuals with any hematological malignancy, compared to controls.

Several cohort studies have investigated the association between lipid fractions and risk of hematological malignancy. Most, but not all, of them reported an inverse association between level of TC [25, 26, 29, 40, 41], LDL-C [25, 29], and HDL-C [25, 27, 29, 31, 42] and risk of any hematological malignancy, in line with our results. In contrast, other studies reported no altered risk of any hematological malignancy in relation to these lipid fractions [32, 43]. Several of these studies demonstrated a lack of association of TG with any hematological malignancy [25, 26]. In our study, although an association was not noted when studying TG per SD increase or comparing the higher quartiles to the lowest quartile, we did observe a positive association between clinically abnormally high TG and a higher risk of hematological malignancy. Future studies are therefore needed to validate the latter finding. Focusing on individual hematological malignancies, we observed an inverse association of TC and LDL-C with multiple myeloma, in line with two previous studies [29, 44]. We also found an inverse association between TC and non-Hodgkin lymphoma, in contrast to one previous study [44]. A lower risk of NHL in relation to a higher level of TG, as noted in the present study, is in agreement with a previous study [45]. In addition, we found a lower risk of acute myeloid leukemia in relation to a higher level of HDL-C, corroborating a previous study [42]. 

In distinction to lipid fractions, fewer cohort studies have focused on the associations of glucose and apolipoproteins with the risk of hematological malignancy [24, 26, 27]. We observed no association between glucose and any hematological malignancy, in accordance with two previous studies [24, 26]. One previous study demonstrated an inverse association between ApoA-I and risk of hematological malignancy [27], in agreement with our findings. To the best of our knowledge, there is no previous cohort study on the risk of hematological malignancy in relation to ApoB. Our finding of a null association between ApoB and risk of hematological malignancy awaits to be validated in future studies, therefore.

There are several potential reasons that could explain the conflicting findings, particularly for lipid fractions, among the previous cohort studies as well as between the previous cohort studies and the present study. The first potential reason is reverse causality, namely that serum metabolic biomarkers might be secondary to hematological malignancy. Previous cohort studies either did not use a lag time or used a relatively short lag time in the analysis [22–33]. In the present study, we focused on the first measurement of metabolic biomarkers during the recruitment period of the AMORIS cohort and excluded the first five years of follow-up in the prospective time-to-event analysis, relieving such concern to the maximum degree. Persistently lower levels of TC, LDL-C, HDL-C and ApoA-I observed more than 20 years before diagnosis of hematological malignancy further argued against reverse causality as an important explanation to our findings. The second potential reason might be difference in the statistical modeling of the metabolic biomarkers. Some studies modeled metabolic biomarkers as categorical variables using quintile or specific cutoffs whereas others fitted metabolic biomarkers as continuous variables using per SD or per unit change. In the present study, we performed analyses using both continuous representation and categorical classification of the biomarkers in separate analyses and observed very similar result patterns. In the classification according to clinical cutoffs, we additionally observed a positive association of TG with risk of Hodgkin lymphoma and chronic lymphoid leukemia, indicating that some difference in the observed association might be due to various analytical approaches. Moreover, the fact that important confounders were not always well controlled in some of the previous studies might also contribute to the contrasting findings. In the present study, we adjusted for several covariates to reduce potential confounding, in the main or sensitivity analyses, including factors related to inflammation, diabetes, smoking, and BMI. Besides, as the metabolic biomarkers are correlated with each other to different extent [35, 36, 46, 47], we also adjusted the studied metabolic biomarkers for one another in a sensitivity analysis to understand the independent roles of the individual biomarkers. Finally, different sample sizes might have led to differential statistical power between studies in disclosing a real association whereas it remains to be examined whether there are biological differences between populations of different background (e.g., Europeans and Asians) could also have contributed to the inconsistent findings.

The mechanisms underlying the association between lipids and hematological malignancies are currently not well understood. Inflammatory pathways might play a crucial role in the development of hematological malignancy [48]. For instance, HDL-C has antioxidant and anti-inflammatory effect [49] whereas ApoA-I is the major protein component of HDL-C. A higher level of HDL-C or Apo A-I may reduce inflammation and cell proliferation signaling pathways [50]. Low levels of HDL-C or ApoA-I can cause inadequate anti-inflammatory response, perhaps leading to abnormal bone marrow proliferation and leukocytosis [51]. In addition, insufficient level of cholesterol might affect activation and function of immune cells (such as macrophage and T cell), which might further contribute to the pathogenesis of hematological malignancies [52]. Low level of LDL might induce increased production of LDL receptor (LDLR) in blood cells through reduced negative feedback, which might further contribute to proliferation of malignant cells [53]. However, the possibility that reduced level of these lipids might be the consequence of special metabolism of precancerous blood cells in very early stage could not be excluded entirely.

The major merit of this study is the large sample size and complete follow-up of more than 30 years, reducing concern on random error due to lack of statistical power, selection bias due to selective study participation or loss to follow-up, as well as systemic bias due to reverse causation. In addition, the prospective and independent collection of data on the studied biomarkers and diagnosis of hematological malignancy minimized potential risk of information bias. Moreover, the detailed adjustment for multiple potential confounders, including infection and autoimmune disorders as well as inflammatory biomarkers, reduced concern on confounding, e.g., due to inflammation. Our study also has limitations. One major limitation is that, given its observational nature, we cannot conclude the observed associations as causal. In addition, as participants in the study were mainly born in Sweden, the generalization of our results to other populations due to different prevalence of hematological malignancy, levels of the studied biomarkers, or genetic and environmental background, needs further investigation.

In summary, we observed a lower risk of hematological malignancy in relation to a higher level of TC, LDL-C, HDL-C, and ApoA-I. Specifically, we noted an inverse association of HDL-C with acute myeloid leukemia, of TC and LDL-C with multiple myeloma, and of TC, TG and ApoA-I with non-Hodgkin lymphoma. Our findings add novel evidence to the role of metabolic factors in the development of hematological malignancy and call for clinical awareness of potential hematological malignancy among individuals with lower levels of lipid fractions and ApoA-I.

## Electronic supplementary material

Below is the link to the electronic supplementary material.

Supplementary Material 1
