# Supplementary material for: Lipids, apolipoproteins, carbohydrates, and risk of hematological malignancies
Source: Eur J Epidemiol. 2025 Mar 4;40(2):187–96. doi: 10.1007/s10654-025-01207-y (PMC12018483; doi:10.1007/s10654-025-01207-y)
Supplement: Supplementary file 1 — Supplementary Material 1 [file 10654_2025_1207_MOESM1_ESM.pdf]

Supplementary material

Table S1. **Summary of existing studies on metabolic biomarkers and the risk of hematological malignancies**

Table S2. **ICD codes for any hematological malignancy and subtypes of hematological malignancy**

Table S3. **ICD codes for autoimmune disorders and hospital-treated infections**

Table S4. **Hazard ratios (HRs) with 95% confidence intervals (CIs) of any hematological malignancy per standard deviation increase of blood biomarkers of lipid, carbohydrate, and apolipoprotein metabolism, analyses further adjusted for history of autoimmune disorders, history of hospital-treated infections, blood level of albumin, blood level of haptoglobin, or blood level of C-reaction protein**

Table S5. **Correlations among the studied metabolic biomarkers**

Table S6. **Hazard ratios (HRs) with 95% confidence intervals (CIs) of any hematological malignancy per standard deviation increase of blood biomarkers of lipid, carbohydrate, and apolipoprotein metabolism, analyses further adjusted for other metabolic biomarkers**

Table S7. **Hazard ratios (HRs) with 95% confidence intervals (CIs) of hematological malignancy per standard deviation increase of blood biomarkers of lipid, carbohydrate, and apolipoprotein metabolism, analyses restricted to first blood collection in relation to an occupational health check-up**

Table S8. **Hazard ratios (HRs) with 95% confidence intervals (CIs) of hematological malignancy per standard deviation increase of blood biomarkers of lipid, carbohydrate, and apolipoprotein metabolism, analyses adjusted for multiple comparison using Bonferroni correction**

Table S9. **Hazard ratios (HRs) with 95% confidence intervals (CIs) of hematological malignancy in relation to different levels of blood biomarkers of lipid, carbohydrate, and apolipoprotein metabolism, analyses using the clinical cutoff values of the biomarkers**

Table S10. Hazard ratios (HRs) with 95% confidence intervals (CIs) of any hematological malignancy per standard deviation increase of blood biomarkers of lipid, carbohydrate, and apolipoprotein metabolism, analyses further adjusted for diagnosis of diabetes, BMI, or smoking

Table S1. Summary of existing studies on metabolic biomarkers and the risk of hematological malignancies

Study; country	Cancer type	Biomarker; outcome	Study design	Sample size	Result
Glucose					
Nagel et al. (2012)[1]; Austria, Norway, Sweden	Hematological malignancies (HM), including acute myeloid leukemia (AML), Non-Hodgkin's lymphoma (NHL), Hodgkin's lymphoma (HL), and multiple myeloma (MM)	Glucose; relative risk (RR)	Cohort study with 13.3 years of follow-up for male, and 11.1 years of follow-up for female	578,700 cohort participants with 2,751 incident cases of HM	Male: RR=1.00 (95%CI=0.85-1.19) per 1 mmol/l increase in glucose; Female: RR=1.16 (95%CI=0.94-1.43) per 1 mmol/l increase in glucose
Stocks et al. (2009)[2]; Austria, Norway, Sweden	HM	Glucose; relative risk (RR)	Cohort with 10.4 years of follow-up	549,944 cohort participants with 1,426 incident cases of HM	Male: No altered risk per quintile increase in glucose (P for trend: 0.3); RR=1.10 (95%CI=0.95-1.28) per 1 mmol/l increase Female: No altered risk per quintile increase in glucose (P for trend: 0.3); RR=1.19 (95%CI=0.99-1.43) per 1 mmol/l increase
Total cholesterol (TC)					
Jung et al. (2022)[3]; Korea	HM, including MM, HL, NHL, AML, and acute lymphoid leukemia (ALL)	TC; hazard ratio (HR)	Cohort study with 8.4 years of follow-up	2,811,410 cohort participants with 5,449 incident cases of HM	Decreased risk per quartile increase; P for trend: <0.001
Loosen et al. (2022)[4]; Germany	HM	TC; odds ratio (OR)	Nested case-control study	7,526 cases and 7,526 sex- and age-matched controls	200-250 mg/dl vs <200 mg/dl: OR=0.91(95%CI=0.81-1.03) >250 mg/dl vs. <200 mg/l: OR=0.85 (95%CI=0.69-1.05)
Fortuny et al. (2022)[5]; United Kingdom	Lymphoma, including NHL and HL	Serum trajectories of TC in the years before diagnosis	Nested case-control study	NHL: 11,803 cases and 57,974 controls HL: 468 cases and 2,302 controls	Mean TC level decreased in the 4 years before diagnosis of lymphoma
Choi et al. (2021)[6]; Korea	MM	TC; hazard ratio (HR)	Cohort study with 5.1 years of follow-up	3,527,776 cohort participants with 969 incident cases of MM	Decreased risk per quartile increase;
Ozturk et al. (2021)[7]; Turkey	HM	TC; mean difference between groups	Cross-sectional study	98 cases and 40 controls	153 mg/dl among cases with HM vs. 196.7 mg/dl among controls; P for difference: <0.001

Guan et al. (2018)[8]; China	Leukemia/lymphoma	TC; hazard ratio (HR)	Cohort study with 8 years of follow-up	68,759 cohort participants with 91 incident cases of lymphoma/leukemia	No altered risk per quartile increase; P for trend: 0.364
Yavasoglu et al. (2017)[9]; Turkey	Chronic lymphoid leukemia (CLL)	TC; mean difference between groups	Cross-sectional study	560 cases and 71 controls	175 mg/dl among cases vs. 217 mg/dl among controls; P for difference: <0.001
Usman et al. (2015)[10]; Germany	Acute leukemia	TC; mean difference between groups	Cross-sectional study	57 cases (25 with AML, 32 with ALL) with 70 controls	P for difference: >0.05
Çebi et al. (2015)[11]; Turkey	AML	TC; mean difference between groups	Cross-sectional study	16 cases and 16 controls	181.8 mg/dl among cases vs. 163 mg/dl among controls; P for difference: 0.37
Strohmaier et al. (2013)[12]; Norway, Austria, Sweden	HM	TC; hazard ratio (HR)	Cohort study (Norwegian Counties study cohort with 25.8 years of follow-up, Oslo cohort with 26 years of follow-up, CONOR cohort with 6.1 years of follow-up)	577,330 cohort participants with 1,790 incident cases of HM	Male: Decreased risk per quintile increase (P for trend: 0.02); HR=0.91 (95%CI=0.85-0.97) per 1 mmol/l increase<br>Female: Decreased risk per quintile increase (P for trend: 0.01); HR=0.85 (95%CI=0.78-0.92) per 1 mmol/l increase
Nagel et al. (2012)[1]; Austria, Norway, Sweden	HM, including AML, NHL, HL, MM	TC; relative risk (RR)	Cohort study with 13.3 years of follow-up for male, and 11.1 years of follow-up for female	133,820 cohort participants with 2,751 incident cases of HM	Male: RR=0.90 (95%CI=0.85-0.95) per 1 mmol/l increase<br>Female: RR=0.88 (95%CI=0.80-0.97) per 1 mmol/l increase
Mulas et al. (2011)[13]; Italy	Lymphocytic leukemia	TC; mean difference between groups	Cross-sectional study	30 cases and 15 age-matched controls	150 mg/dl among cases vs. 172 mg/dl among controls; P for difference: 0.30
Strasak et al. (2009)[14]; Austria	HM	TC; hazard ratio (HR)	Cohort study with ≥10 years of follow-up	172,210 cohort participants with 644 cases of HM	Male: No altered risk per tertile increase (P for trend: 0.71)<br>Female: No altered risk per tertile increase (P for trend: 0.12)
Iso et al. (2009)[15]; Japan	Leukemia	TC; hazard ratio (HR)	Cohort study with 12.4 years of follow-up	33,368 cohort participants with 50 incident cases of leukemia	Per standard deviation increase of TC: HR=1.16 (95%CI=0.78-1.71)

Ahn et al. (2009)[16]; United States	HM	TC; relative risk (RR)	Cohort study with 18 years of follow-up	29,093 cohort participants with 324 incident cases of HM	No altered risk per quintile increase; P for trend: 0.22
Yavasoglu et al. (2008)[17]; Turkey	MM	TC; mean difference between groups	Cross-sectional study	102 cases with 71 controls	159 mg/dl among cases vs. 217 mg/dl among controls; P for difference: <0.001
Kuliszkiewicz-Janus et al. (2008)[18]; Poland	HM, including ALL, NHL, HL, MM	TC; mean difference between groups	Cross-sectional study	238 cases with 110 controls	Cases with AL or NHL had lower LDL-C level compared to controls; P for difference: <0.05
Naik et al. (2006)[19]; India	Leukemia, HL	TC; mean difference between groups	Cross-sectional study	105 cases and 52 controls	Leukemia: 131.36 mg/dl among cases vs. 153.86 mg/dl (P for difference: <0.001)<br>HL: 139.4 mg/dl among cases vs. 153.86 mg/dl among controls (P for difference: <0.001)
Goncalves et al. (2005)[20]; Brazil	AML, ALL	TC; mean difference between groups	Cross-sectional study	12 cases with 6 controls	ALL: 152.6 mg/dl among cases vs. 181.0 mg/dl among controls (P for difference: <0.05)<br>AML: 113.2 mg/dl among cases vs. 181.0 mg/dl among controls (P for difference: <0.001)
Fiorenza et al. (2000)[21]; Italy	HM	TC; mean difference between groups	Cross-sectional study	97 cases and 97 controls	157.1 mg/dl among cases vs. 206.6 mg/dl among controls; P for difference: <0.05
Halton et al. (1998)[22]; Canada	ALL	TC; mean difference between groups	Cross-sectional study	24 cases and 15 controls	3.49 mmol/l among cases vs. 3.66 mmol/l among controls; P for difference: >0.05
Baroni et al. (1996)[23]; Italy	ALL and acute nonlymphocyte leukemia (ANLL)	TC; mean difference between groups	Cross-sectional study	18 cases of ALL and 25 cases of ANLL, and 25 controls	ALL: 150.0 mg/dl among cases vs. 223.1 mg/dl among controls (P for difference: <0.001)<br>ANLL: 141.0 mg/dl among cases vs. 223.1 mg/dl among controls (P for difference: <0.001)
Baroni et al. (1994)[24]; Italy	ALL	TC; mean difference between groups	Cross-sectional study	10 cases and 10 controls	140.5 mg/dl among cases vs. 213.0 mg/dl among controls; P for difference: <0.001
Chyou et al. (1992)[25]; United States	Leukemia, lymphoma	TC; hazard ratio (HR)	Cohort study with 19 years of follow-up	7716 cohort participants with 73 (32 leukemia, 41 lymphoma) incident cases of HM	No significant association between TC level and the risk of leukemia/lymphoma

Knekt et al. (1988)[26]; Finland	HM	TC; relative risk (RR)	Cohort study with 10 years of follow-up	39,268 cohort participants with 45 cases of HM	Male: Decreased risk per tertile increase (P for trend: 0.019) Female: Decreased risk with increased TC level (P for trend: 0.259)
Hiatt et al. (1986)[27]; United States	Lymphoma	TC; relative risk (RR)	Cohort study with 10 years of follow-up	160,135 cohort participants with 283 incident cases of lymphoma	RR=1.61 (95%CI=1.01-2.58) among males comparing <185 mg/dl to ≥185 mg/dl
LDL-C					
Jung et al. (2022)[3]; Korea	HM, including MM, HL, NHL, ALL, AML	LDL-C; hazard ratios (HR)	Cohort study with 8.4 years of follow-up	2,811,410 cohort participants with 5,449 incident cases of HM	Decreased risk per quartile increase; P for trend: <0.001
Loosen et al. (2022)[4]; Germany	HM	LDL-C; odds ratio (OR)	Nested case-control study	7,526 cases and 7,526 sex- and age-matched controls	100-160 mg/dl vs <100 mg/dl: OR=0.94 (95%CI=0.82-1.08) >160 mg/dl vs. <100 mg/dl: OR=0.93 (95%CI=0.75-1.14)
Fortuny et al. (2022)[5]; United Kingdom	Lymphoma including NHL and HL	Serum trajectories of LDL-C in the years before diagnosis	Nested case-control study	NHL: 7,858 cases and 31,801 controls HL: 302 cases and 1,231 controls	Mean LDL-C level decreased in the 4 years before diagnosis of lymphoma
Choi et al. (2021)[6]; Korea	MM	LDL-C; hazard ratio (HR)	Cohort study with 5.1 years of follow-up	3,527,776 cohort participants with 969 incident cases of MM	Decreased risk per quartile increase
Ozturk et al. (2021)[7]; Turkey	HM	LDL-C; mean difference between groups	Cross-sectional study	98 cases and 40 controls	89 mg/dl among cases with HM vs. 118.5 mg/dl among controls; P for difference: <0.001
Guan et al. (2018)[8]; China	Leukemia/lymphoma	LDL-C; hazard ratios (HR)	Cohort study with 8 years of follow-up	68,759 cohort participants with 91 incident cases of lymphoma/leukemia	No altered risk per quartile increase; P for trend: 0.539
Yavasoglu et al. (2017)[9]; Turkey	CLL	LDL-C; mean difference between groups	Cross-sectional study	560 cases and 71 controls	107 mg/dl among cases vs. 131 mg/dl among controls; P for difference: <0.001
Usman et al. (2014)[10]; Germany	Acute leukemia	LDL-C; mean difference between groups	Cross-sectional study	57 cases and 70 controls	P for difference: >0.05

Çebi et al. (2015)[11]; Turkey	AML	LDL-C; mean difference between groups	Cross-sectional study	16 cases and 16 controls	120.6 mg/dl among cases vs. 92 mg/dl among controls; P for difference: 0.12
wang et al. (2014)[28]; China	Lymphoma, extranodal natural killer/T cell lymphoma, nasal type	LDL-C; mean difference between groups	Cross-sectional study	107 cases and 430 controls	2.85 mmol/l among cases vs. 2.91 mmol/l among controls; P for difference: >0.05
Yavasoglu et al. (2008)[17]; Turkey	MM	LDL-C; mean difference between groups	Cross-sectional study	102 cases and 71 controls	82.5 mg/dl among cases vs. 131 mg/dl among controls; P for difference: <0.001
Yang et al. (2008)[29]; China	Leukemia	LDL-C; hazard ratios (HR)	Cohort study with 4.90 years of follow-up	6,107 cohort participants with type 2 diabetes mellitus, including 8 incident cases of leukemia	<2.8 mmol/L vs. 2.8-3.8 mmol/L: HR=6.39 (95%CI=0.55-74.64) >3.8 mmol/L vs. 2.8-3.8 mmol/L: HR=4.76 (95%CI=0.43-52.56)
Kuliszkiewicz-Janus et al. (2008)[18]; Poland	HM, including ALL, NHL, HL, MM	LDL-C; mean difference between groups	Cross-sectional study	238 cases and 110 controls	Cases had lower level than controls; P for difference: <0.05
Naik et al. (2006)[19]; India	Leukemia, HL	LDL-C; mean difference between groups	Cross-sectional study	105 cases (55 with leukemia, 50 with NL) and 52 controls	Leukemia: 77.35 mg/dl among cases vs. 104.54 mg/dl among controls (P for difference: <0.001) HL: 86.5 mg/dl among cases vs. 104.54 mg/dl among controls (P for difference: <0.001)
Goncalves et al. (2005)[20]; Brazil	AML, ALL	LDL-C; mean difference between groups	Cross-sectional study	12 cases and 6 controls	ALL: 84.0 mg/dl among cases vs. 108.2 mg/dl among controls (P for difference: >0.05) AML: 40.5 mg/dl among cases vs. 108.2 mg/dl among controls (P for difference: <0.01)
Fiorenza et al. (2000)[21]; Italy	HM	LDL-C; mean difference between groups	Cross-sectional study	97 cases and 97 age- and sex-matched controls	92.3 mg/dl among cases vs. 125.5 mg/dl among controls; P for difference: <0.05
Halton et al. (1998)[22]; Canada	ALL	LDL-C; mean difference between groups	Cross-sectional study	24 cases and 15 controls	2.05 mmol/l among cases vs. 1.98 mmol/l among controls; P for difference: >0.05
Baroni et al. (1996)[23]; Italy	ALL and acute nonlymphocyte	LDL-C; mean	Cross-sectional study	18 cases of ALL and 25 cases of	ALL: 85.4 mg/dl among cases vs. 140.4 mg/dl

	leukemia (ANLL)	difference between groups		ANLL, and 25 controls	among controls (P for difference: <0.001)<br>ANLL: 77.0 mg/dl among cases vs. 140.4 mg/dl among controls (P for difference: <0.001)
Baroni et al. (1994)[24]; Italy	Acute lymphoblastic leukemia	LDL-C; mean difference between groups	Cross-sectional study	10 cases and 10 controls	29.12 mg/dl among cases vs. 55.8 mg/dl among controls; P for difference: <0.001
HDL-C					
Jung et al. (2022)[3]; Korea	HM, including MM, HL, NHL, ALL, AML	HDL-C; hazard ratios (HR)	Cohort study with 8.4 years of follow-up	2,811,410 cohort participants with 5,449 incident cases of HM	Decreased risk per quartile increase; P for trend: <0.001
Loosen et al. (2022)[4]; Germany	HM	HDL-C; odds ratios (OR)	Nested case-control study	7,526 cases and 7,526 sex- and age-matched controls	31-35 mg/dl vs >40 mg/dl: OR=1.16 (95%CI=0.98-1.36)<br><35 mg/dl vs. >40 mg/dl: OR=1.27 (95%CI=1.04-1.54)
Fortuny et al. (2022)[5]; United Kingdom	Lymphoma including NHL and HL	HDL-C; Serum trajectories in the years before diagnosis	Nested case-control study	NHL: 9,391 cases and 40,667 controls<br>HL: 357 cases and 1,574 controls	Mean HDL-C level decreased in the 4 years before diagnosis of lymphoma
Choi et al. (2021)[6]; Korea	MM	HDL-C; hazard ratio (HR)	Cohort study with 5.1 years of follow-up	3,527,776 cohort participants with 969 incident cases of MM	Decreased risk per quartile increase
Ozturk et al. (2021)[7]; Turkey	HM	HDL-C; mean difference between groups	Cross-sectional study	98 cases and 40 controls	29 mg/dl among cases vs. 49 mg/dl among controls; P for difference: <0.001
Jeong et al. (2021)[30]; Korea	HM, including MM, HL, NHL, ALL, AML	HDL-C; hazard ratio (HR)	Cohort study with 8.3 years of follow-up	9,596,145 cohort participants with 15,864 incident cases of HM	Increased risk per quartile decrease
Pedersen et al. (2020)[31]; Denmark	HM	HDL-C; hazard ratio (HR)	Cohort study with 18 years of follow-up	106,856 cohort participants with 1,007 incident cases of HM	Lower HDL-C level associated with increased risk; P for trend: 6x10^{-6}
Yavasoglu et al. (2017)[9]; Turkey	CLL	HDL-C; mean difference between groups	Cross-sectional study	560 cases and 71 controls	37 mg/dl among cases vs. 53 mg/dl among controls; P for difference: <0.001

Usman et al. (2015)[10]; Germany	Acute leukemia	HDL-C; mean difference between groups	Cross-sectional study	57 cases (25 with AML, 32 with ALL) and 70 controls	Lower level of HDL-C among cases vs. controls; P for difference: <0.05
Çebi et al. (2015)[11]; Turkey	AML	HDL-C; mean difference between groups	Cross-sectional study	16 cases and 16 controls	31.5 mg/dl among cases vs. 43.7 mg/dl among controls; P for difference: 0.002
wang et al. (2014)[28]; China	Lymphoma, extranodal natural killer/T cell lymphoma, nasal type	HDL-C; mean difference between groups	Cross-sectional study	107 cases and 430 controls	1.10 mmol/l among cases vs. 1.38 mmol/l among controls; P for difference: <0.05
Mulas et al. (2011)[13]; Italy	Lymphocytic leukemia	HDL-C; mean difference between groups	Cross-sectional study	30 cases and 15 age-matched controls	30 mg/dl among cases vs. 52 mg/dl among controls; P for difference: <0.001
Ahn et al. (2009)[16]; United States	HM	HDL-C; relative risk (RR)	Cohort study with 18 years of follow-up	29,093 cohort participants with 324 incident cases of HM	Decreased risk per quintile (until 4 quintile) increase; P for trend: 0.03
Yavasoglu et al. (2008)[17]; Turkey	MM	HDL-C; mean difference between groups	Cross-sectional study	102 cases and 71 controls	42 mg/dl among cases vs. 53 mg/dl among controls; P for difference: <0.001
Kuliszkiewicz-Janus et al. (2008)[18]; Poland	HM (AL, NHL, HL, MPS, MM)	HDL-C; mean difference between groups	Cross-sectional study	238 cases and 110 controls	Cases had lower HDL-C level than controls; P for difference: <0.05
Lim et al. (2007)[32]; United States	NHL	HDL-C; relative risk (RR)	Cohort study with 17.4 years of follow-up	27,074 cohort participants with 201 cases of NHL	Early follow-up (<10 years): decreased risk per quintile increase; P for trend: <0.001
Naik et al. (2006)[19]; India	Leukemia, HL	HDL-C; mean difference between groups	Cross-sectional study	105 cases (55 with leukemia, 50 with HL) and 52 controls	Leukemia: 22.76 mg/dl among cases vs. 35.76 mg/dl (P for difference: <0.001)<br>HL: 27.78 mg/dl among cases vs. 35.76 mg/dl among controls (P for difference: <0.001)
Goncalves et al. (2005)[20]; Brazil	AML, ALL	HDL-C; mean difference between groups	Cross-sectional study	12 cases (6 ALL and 6 AML) and 6 controls	ALL: 19.4 mg/dl among cases vs. 47.0 mg/dl among controls (P for difference: <0.01)<br>AML: 25.3 mg/dl among cases vs. 47.0 mg/dl among controls (P for difference: <0.05)

Fiorenza et al. (2000)[21]; Italy	HM	HDL-C; mean difference between groups	Cross-sectional study	97 cases and 97 age- and sex-matched controls	37.7 mg/dl among cases vs. 55.6 mg/dl among controls; P for difference: <0.05
Halton et al. (1998)[22]; Canada	ALL	HDL-C; mean difference between groups	Cross-sectional study	24 cases and 15 controls	0.54 mmol/l among cases vs. 1.28 mmol/l among controls; P for difference: <0.05
Baroni et al. (1996)[23]; Italy	ALL and acute nonlymphocyte leukemia (ANLL)	HDL-C; mean difference between groups	Cross-sectional study	18 cases of ALL and 25 cases of ANLL, and 25 controls	ALL: 27.3 mg/dl among cases vs. 60.3 mg/dl among controls (P for difference: <0.001) ANLL: 37.0 mg/dl among cases vs. 60.3 mg/dl among controls (P for difference: <0.001)
Baroni et al. (1994)[24]; Italy	ALL	HDL-C; mean difference between groups	Cross-sectional study	10 cases and 10 controls	P for difference: >0.05
Triglycerides (TG)					
Jung et al. (2022)[3]; Korea	HM, including MM, HL, NHL, ALL, AML	TG; hazard ratio (HR)	Cohort study with 8.4 years of follow-up	2,811,410 cohort participants with 5,449 incident cases of HM	No altered risk per quartile increase; P for trend: 0.352
Loosen et al. (2022)[4]; Germany	HM	TG; odds ratio (OR)	Nested case-control study	7,526 cases and 7,526 sex- and age-matched controls	150-200 mg/dl vs <150 mg/dl: OR=0.90 (95%CI=0.80-1.01) >200 mg/dl vs. <150 mg/dl: OR=0.99 (95%CI=0.86-1.13)
Fortuny et al. (2022)[5]; United Kingdom	Lymphoma, including NHL and HL	TG; Serum trajectories in the years before diagnosis	Nested case-control study	NHL: 4,090 cases and 10,969 controls HL: 140 cases and 396 controls	No difference between cases and controls
Choi et al. (2021)[6]; Korea	MM	TG; hazard ratio (HR)	Cohort study with 5.1 years of follow-up	3,527,776 cohort participants with 969 incident cases of MM	Decreased risk per quartile increase
Ozturk et al. (2021)[7]; Turkey	HM	TG; mean difference between groups	Cross-sectional study	98 cases and 40 controls	179.5 mg/dl among cases vs. 145.8 mg/dl among controls; P for difference: 0.02
Yavasoglu et al. (2017)[9]; Turkey	CLL	TG; mean difference between groups	Cross-sectional study	560 cases and 71 controls	140 mg/dl among cases vs. 147 mg/dl among controls; P for difference: >0.05

Usman et al. (2015)[10]; Germany	Acute leukemia	TG; mean difference between groups	Cross-sectional study	57 cases (25 with AML, 32 with ALL) and 70 controls	Higher level of TG in cases vs. controls; P for difference: <0.001
Çebi et al. (2015)[11]; Turkey	AML	TG; mean difference between groups	Cross-sectional study	16 cases and 16 controls	157.3 mg/dl among cases vs. 136.2 mg/dl among controls; P for difference: 0.46
Nagel et al. (2012)[1]; Austria, Norway, Sweden	HM, including AML, NHL, HL, MM	TG; relative risk (RR)	Cohort study with 13.3 years of follow-up for male, and 11.1 years of follow-up for female	133,820 cohort participants with 2,751 incident cases of HM	Male: RR=1.00 (95%CI=0.91-1.10) per 1 mmol/l increase<br>Female: RR=0.93 (95%CI=0.82-1.06) per 1 mmol/l increase
Mulas et al. (2011)[13]; Italy	Lymphocytic leukemia	TG; mean difference between groups	Cross-sectional study	30 cases and 15 age-matched controls	110 mg/dl among cases vs. 88 mg/dl among controls; P for difference: 0.15
Ulmer et al. (2009)[33]; Austria	NHL	TG; hazard ratio (HR)	Cohort study	156,153 cohort participants with 219 incident cases of NHL	HR=0.71 (95%CI=0.52-0.97) per log-unit increase
Yavasoglu et al. (2008)[17]; Turkey	MM	TG; mean difference between groups	Cross-sectional study	102 cases and 71 controls	147 mg/dl among cases vs. 149 mg/dl among controls; P for difference: >0.05
Kuliszkiewicz-Janus et al. (2008)[18]; Poland	HM, including AL, NHL, HL, MM	TG; mean difference between groups	Cross-sectional study	238 cases and 110 controls	Cases had higher level than controls; P for difference: <0.05
Naik et al. (2006)[19]; India	Leukemia, HL	TG; mean difference between groups	Cross-sectional study	105 cases (55 with leukemia, 50 with HL) and 52 controls	Leukemia: 167.12 mg/dl among cases vs. 73.9 mg/dl (P for difference: <0.001)<br>HL: 143.9 mg/dl among cases vs. 73.9 mg/dl among controls (P for difference: <0.001)
Goncalves et al. (2005)[20]; Brazil	AML, ALL	TG; mean difference between groups	Cross-sectional study	12 cases and 6 controls	ALL: 182.2 mg/dl in cases vs. 133.8 mg/dl in controls (P for difference: >0.05)<br>AML: 197.5 mg/dl in cases vs. 133.8 mg/dl in controls (P for difference: >0.05)
Fiorenza et al. (2000)[21]; Italy	HM	TG; mean difference between groups	Cross-sectional study	97 cases and 97 age- and sex-matched controls	135.7 mg/dl among cases vs. 127.4 mg/dl among controls; P for difference: <0.05
Halton et al. (1998)[22]; Canada	ALL	TG; mean difference between	Cross-sectional study	24 cases and 15 controls	1.82 mmol/l among cases vs. 0.88 mmol/l among controls; P for difference:

		groups			<0.05
Baroni et al. (1996)[23]; Italy	ALL and acute nonlymphocyte leukemia (ANLL)	TG; mean difference between groups	Cross-sectional study	18 cases of ALL and 25 cases of ANLL, and 25 controls	ALL: 201.0 mg/dl in cases vs. 117.7 mg/dl in controls (P for difference: <0.001)<br>ANLL: 134.0 mg/dl in cases vs. 117.7 mg/dl in controls (P for difference: >0.05)
Baroni et al. (1994)[24]; Italy	ALL	TG; mean difference between groups	Cross-sectional study	10 cases and 10 controls	P for difference: >0.05
Apolipoprotein A-I (ApoA-I)					
Pedersen et al. (2020)[31]; Denmark	HM	ApoA-I; hazard ratio (HR)	Cohort study with 18 years of follow-up	106,856 cohort participants with 1,007 incident cases of HM	Lower ApoA-I level associated with increased risk; P for trend: 8×10^{-6}
Zhang et al. (2015)[34]; China	MM	ApoA-I; mean difference between groups	Cross-sectional study	8 cases and 8 controls	Higher level of ApoA-I in cases vs. controls
Halton et al. (1998)[22]; Canada	ALL	ApoA-I; mean difference between groups	Cross-sectional study	24 cases and 15 controls	0.77 g/l among cases vs. 1.22 g/l among controls; P for difference: <0.05
Baroni et al. (1996)[23]; Italy	ALL and acute nonlymphocyte leukemia (ANLL)	ApoA-I; mean difference between groups	Cross-sectional study	18 cases of ALL and 25 cases of ANLL, and 25 controls	ALL: 79.7 mg/dl in cases vs. 146.3 mg/dl in controls (P for difference: <0.001)<br>ANLL: 91.0 mg/dl in cases vs. 146.3 mg/dl in controls (P for difference: <0.001)
Baroni et al. (1994)[24]; Italy	ALL	ApoA-I; mean difference between groups	Cross-sectional study	10 cases and 10 controls	P for difference: >0.05
Apolipoprotin B (ApoB)					
Zhang et al. (2015)[34]; China	MM	ApoB; mean difference between groups	Cross-sectional study	8 cases and 8 controls	Lower level of ApoB in cases vs. controls
Halton et al. (1998)[22]; Canada	ALL	ApoB; mean difference between groups	Cross-sectional study	24 cases and 15 controls	1.06 g/l among cases vs. 0.80 g/l among controls; P for difference: <0.05

Baroni et al. (1996)[23]; Italy	ALL and acute nonlymphocyte leukemia (ANLL)	ApoB; mean difference between groups	Cross-sectional study	18 cases of ALL and 25 cases of ANLL, and 25 controls	ALL: 92.8 mg/dl in cases vs. 105.1 mg/dl in controls (P for difference: >0.05) ANLL: 82.0 mg/dl in cases vs. 105.1 mg/dl in controls (P for difference: <0.001)
Baroni et al. (1994)[24]; Italy	ALL	ApoB; mean difference between groups	Cross-sectional study	10 cases and 10 controls	P for difference: >0.05

Reference

1 Nagel, G. *et al.* Metabolic factors and blood cancers among 578,000 adults in the metabolic syndrome and cancer project (Me-Can). *Ann Hematol* **91**, 1519-1531 (2012). https://doi.org:10.1007/s00277-012-1489-z

2 Stocks, T. *et al.* Blood glucose and risk of incident and fatal cancer in the metabolic syndrome and cancer project (me-can): analysis of six prospective cohorts. *PLoS Med* **6**, e1000201 (2009). https://doi.org:10.1371/journal.pmed.1000201

3 Jung, W. *et al.* Association between Cholesterol Level and the Risk of Hematologic Malignancy According to Menopausal Status: A Korean Nationwide Cohort Study. *Biomedicines* **10** (2022). https://doi.org:10.3390/biomedicines10071617

4 Loosen, S. H., Kostev, K., Luedde, M., Luedde, T. & Roderburg, C. Low blood levels of high-density lipoprotein (HDL) cholesterol are positively associated with cancer. *J Cancer Res Clin Oncol* **148**, 3039-3046 (2022). https://doi.org:10.1007/s00432-021-03867-1

5 Fortuny, J., Plana, E. & Kaye, J. A. Serum lipid trajectories in the years before a lymphoma diagnosis. *Leuk Lymphoma* **63**, 291-303 (2022). https://doi.org:10.1080/10428194.2021.1992618

6 Choi, T. *et al.* Lipid Level, Lipid Variability, and Risk of Multiple Myeloma: A Nationwide Population-Based Study of 3,527,776 Subjects. *Cancers (Basel)* **13** (2021). https://doi.org:10.3390/cancers13030540

7 Ozturk, E. The Relationship Between Hematological Malignancy and Lipid Profile. *Medeni Med J* **36**, 146-151 (2021). https://doi.org:10.5222/MMJ.2021.91145

8 Guan, X. M. *et al.* Association of total cholesterol, low-density lipoprotein cholesterol, and non-high-density lipoprotein cholesterol with atherosclerotic cardiovascular disease and cancer in a Chinese male population. *Int J Cancer* **142**, 1209-1217 (2018). https://doi.org:10.1002/ijc.31149

9 Yavasoglu, I. *et al.* Cholesterol Levels in Patients with Chronic Lymphocytic Leukemia. *J Natl Med Assoc* **109**, 23-27 (2017). https://doi.org:10.1016/j.jnma.2016.11.006

10 Usman, H. *et al.* Revisiting the dyslipidemia associated with acute leukemia. *Clin Chim Acta* **444**, 43-49 (2015). https://doi.org:10.1016/j.cca.2015.01.038

11 Cebi, A., Akgun, E., Esen, R., Demir, H. & Cifci, A. The activities of serum paraoxonase and arylesterase and lipid profile in acute myeloid leukemia: preliminary results. *Eur Rev Med Pharmacol Sci* **19**, 4590-4594 (2015).

12 Strohmaier, S. *et al.* Total serum cholesterol and cancer incidence in the Metabolic syndrome and Cancer Project (Me-Can). *PLoS One* **8**, e54242 (2013). https://doi.org:10.1371/journal.pone.0054242

13 Mulas, M. F. *et al.* Cholesterol esters as growth regulators of lymphocytic leukaemia cells. *Cell Prolif* **44**, 360-371 (2011). https://doi.org:10.1111/j.1365-2184.2011.00758.x
14 Strasak, A. M. *et al.* Time-dependent association of total serum cholesterol and cancer incidence in a cohort of 172,210 men and women: a prospective 19-year follow-up study. *Ann Oncol* **20**, 1113-1120 (2009). https://doi.org:10.1093/annonc/mdn736
15 Iso, H. *et al.* Serum cholesterol levels in relation to the incidence of cancer: the JPHC study cohorts. *Int J Cancer* **125**, 2679-2686 (2009). https://doi.org:10.1002/ijc.24668
16 Ahn, J. *et al.* Prediagnostic total and high-density lipoprotein cholesterol and risk of cancer. *Cancer Epidemiol Biomarkers Prev* **18**, 2814-2821 (2009). https://doi.org:10.1158/1055-9965.EPI-08-1248
17 Yavasoglu, I. *et al.* Cholesterol levels in patients with multiple myeloma. *Ann Hematol* **87**, 223-228 (2008). https://doi.org:10.1007/s00277-007-0375-6
18 Kuliszkiewicz-Janus, M., Malecki, R. & Mohamed, A. S. Lipid changes occuring in the course of hematological cancers. *Cell Mol Biol Lett* **13**, 465-474 (2008). https://doi.org:10.2478/s11658-008-0014-9
19 Naik, P. P., Ghadge, M. S. & Raste, A. S. Lipid profile in leukemia and Hodgkin's disease. *Indian J Clin Biochem* **21**, 100-102 (2006). https://doi.org:10.1007/BF02912921
20 Goncalves, R. P., Rodrigues, D. G. & Maranhao, R. C. Uptake of high density lipoprotein (HDL) cholesteryl esters by human acute leukemia cells. *Leuk Res* **29**, 955-959 (2005). https://doi.org:10.1016/j.leukres.2005.01.013
21 Fiorenza, A. M., Branchi, A. & Sommariva, D. Serum lipoprotein profile in patients with cancer. A comparison with non-cancer subjects. *Int J Clin Lab Res* **30**, 141-145 (2000). https://doi.org:10.1007/s005990070013
22 Halton, J. M., Nazir, D. J., McQueen, M. J. & Barr, R. D. Blood lipid profiles in children with acute lymphoblastic leukemia. *Cancer* **83**, 379-384 (1998).
23 Baroni, S. *et al.* Prognostic relevance of lipoprotein cholesterol levels in acute lymphocytic and nonlymphocytic leukemia. *Acta Haematol* **96**, 24-28 (1996). https://doi.org:10.1159/000203710
24 Baroni, S. *et al.* Lipids and lipoproteins in acute lymphoblastic leukaemia (ALL). *Leuk Res* **18**, 643-644 (1994). https://doi.org:10.1016/0145-2126(94)90047-7
25 Chyou, P. H., Nomura, A. M., Stemmermann, G. N. & Kato, I. Prospective study of serum cholesterol and site-specific cancers. *J Clin Epidemiol* **45**, 287-292 (1992). https://doi.org:10.1016/0895-4356(92)90089-6
26 Knekt, P. *et al.* Serum cholesterol and risk of cancer in a cohort of 39,000 men and women. *J Clin Epidemiol* **41**, 519-530 (1988). https://doi.org:10.1016/0895-4356(88)90056-x
27 Hiatt, R. A. & Fireman, B. H. Serum cholesterol and the incidence of cancer in a large cohort. *J Chronic Dis* **39**, 861-870 (1986). https://doi.org:10.1016/0021-9681(86)90034-2
28 Wang, L. *et al.* Low level of high-density lipoprotein cholesterol correlates with poor prognosis in extranodal natural killer/T cell lymphoma. *Tumour Biol* **35**, 2141-2149 (2014). https://doi.org:10.1007/s13277-013-1284-z
29 Yang, X. *et al.* Independent associations between low-density lipoprotein cholesterol and cancer among patients with type 2 diabetes mellitus. *CMAJ* **179**, 427-437 (2008). https://doi.org:10.1503/cmaj.071474
30 Jeong, S. M. *et al.* Association between high-density lipoprotein cholesterol level and risk of hematologic malignancy. *Leukemia* **35**, 1356-1364 (2021). https://doi.org:10.1038/s41375-020-01081-5
31 Pedersen, K. M., Colak, Y., Bojesen, S. E. & Nordestgaard, B. G. Low high-density lipoprotein and increased risk of several cancers: 2 population-based cohort studies including 116,728 individuals. *J Hematol Oncol* **13**, 129 (2020). https://doi.org:10.1186/s13045-020-00963-6

32 Lim, U. *et al.* Serum high-density lipoprotein cholesterol and risk of non-hodgkin lymphoma. *Cancer Res* **67**, 5569-5574 (2007). https://doi.org:10.1158/0008-5472.CAN-07-0212
33 Ulmer, H. *et al.* Serum triglyceride concentrations and cancer risk in a large cohort study in Austria. *Br J Cancer* **101**, 1202-1206 (2009). https://doi.org:10.1038/sj.bjc.6605264
34 Zhang, H. T., Tian, E. B., Chen, Y. L., Deng, H. T. & Wang, Q. T. Proteomic analysis for finding serum pathogenic factors and potential biomarkers in multiple myeloma. *Chin Med J (Engl)* **128**, 1108-1113 (2015). https://doi.org:10.4103/0366-6999.155112

Table S2. ICD codes for any hematological malignancy and subtypes of hematological malignancy

**Disorder**	**ICD-9**	**ICD-O-2**
Any hematological malignancy	200-208, 2733	C81-C96
Acute myeloid leukemia	2050, 2052, 2053, 2060, 2070	C920, C922, C923, C924, C925, C930, C940, C942
Myeloma	203	C90
Hodgkin's lymphoma	2010, 2011, 2012, 2014, 2015, 2016, 2017, 2019	C810, C811, C812, C813, C814, C817, C819
Non-Hodgkin's lymphoma	2020, 2021, 2022, 2023, 2024, 2028, 2029, 2733	C820-C827, C829-C831, C833-C845, C850, C851, C857, C859, C880, C884, C913, C914, C961, C963, C967, C969
Chronic lymphoid leukemia	2041	C911

Table S3 ICD codes for autoimmune disorders and hospital-treated infections

Diagnoses	ICD-9	ICD-10
**Autoimmune disorders**	250, 242A, 242X, 244W, 244X, 245C, 245W, 258B, 099D, 711A, 714A, 714B, 714C, 714D, 719D, 720A, 446A, 446B, 446G, 446E, 446F, 287A, 725, 710A, 710B, 710C, 710D, 710E, 710W, 710X, 136B, 694E, 694F, 694A, 696A, 696B, 704A, 709A, 281A, 283A, 287D, 340, 341A, 357A, 358A, 571G, 555, 556, 579A, 390, 391, 392, 135, 580, 582	E10, E035, E039, E050, E055, E059, E063, E065, E271, E272, E310, M023, M028, M029, M025, M05, M06, M080, M081, M082, M083, M084, M45, M30, M311, M313, M317, D690, M315, M316, M353, M32, M330, M331, M332, M339, M34, M350, M351, M352, L100, L12, L130, L40, L64, L80, D510, D590, D591, D693, G04, G131, G35, G36, G610, G611, G618, G619, G700, K743, K50, K51, K900, I00, I010, I011, I012, I018, I019, I020, I029, D86, N00, N01, N03, N05

**Infections**	000-139, 245A, 254B, 279K, 320, 321, 323, 324, 360A, 370E, 370F, 370X, 372A, 372B, 372C, 372D, 373B, 375D, 376A, 380B,<br><br>380C, 381A, 382A, 382B, 382C, 382D, 382E, 382X, 383, 390, 391, 392, 420, 421, 422, 460, 461, 462, 463, 464, 465, 466A, 466B, 473, 475, 480, 481, 482, 483, 485, 486, 487, 490, 491B, 510, 511B, 513A, 513B, 522E, 522H, 526E, 527D, 528A, 528D, 540A, 540B, 540X, 562A, 562B, 566, 567A, 567B, 567C, 569F, 572A, 575A, 590, 595A, 595B, 595C, 595D, 595X, 597A, 597W, 599A, 611A, 614, 615A, 615X, 616, 634A, 635A, 636A, 637A, 638A, 639A, 646F, 646G, 647, 658E, 659D, 670, 675A, 675B, 675W, 675X, 680A, 681, 682, 683, 684, 685, 686, 711, 727A, 728A, 729E, 730A, 730B, 730C, 730D,	A00-B99, E060, E321, G00, G01, G02, G04, G050, G051, G052, G060, G061, G062, G07, H000, H010, H043, H050, H100, H103, H104, H105, H106, H107, H108, H109, H162 H163, H169, H32, H440, H60, H650, H651, H660, H661, H662, H663, H664, H669, H70, I00, I01, I02, I30, I33, I400, J00, J01, J02, J03, J04, J05, J06, J10, J11, J12, J13, J14, J15, J16, J18, J20, J21, J22, J32, J340, J350, J36,J370, J371, J390, J391, J40, J41, J42, J851, J852, J853, J86, K046, K047, K052, K113, K122, K140, K35, K359, K351, K57, K610, K611, K612, K613, K614, M711,M86, N10, N11, N12, N136, N151, N159,N300, N301, N302, N303, N308, N309, N340, N341, N390, N61, N70, N71, N72, N73, N74, N75, N76, N980, O070, O075, O080, O23, O353, O411, O753, O85, O86, O910, O911, O98, P23, P35, P36, P37, P38, P39, T802, T814, T826, T827, T835, T836, T845, T846, T847, T857, T880, Z21, Z22

	730X, 770A, 771, 790W, 996G, 998F, 999D	

Table S4. Hazard ratios (HRs) with 95% confidence intervals (CIs) of any hematological malignancy per standard deviation increase of blood biomarkers of lipid, carbohydrate, and apolipoprotein metabolism, analyses further adjusted for history of autoimmune disorders, history of hospital-treated infections, blood level of albumin, blood level of haptoglobin, or blood level of C-reaction protein[a]

Biomarker	Further adjustment for history of autoimmune disorders	Further adjustment for history of hospital-treated infections	Further adjustment for albumin	Further adjustment for haptoglobin	Further adjustment for C-reaction protein
	**HR (95% CI)[b]**	**HR (95% CI)[b]**	**HR (95% CI)[b]**	**HR (95% CI)[b]**	**HR (95% CI)[b]**
Glucose	0.99 (0.97-1.02)	1.00 (0.98-1.02)	1.00 (0.97-1.03)	1.01 (0.98-1.03)	0.98 (0.95-1.02)
TC	**0.93 (0.91-0.96)**	**0.93 (0.91-0.96)**	**0.95 (0.92-0.97)**	**0.95 (0.92-0.97)**	**0.91 (0.87-0.95)**
LDL-C	**0.94 (0.91-0.97)**	**0.94 (0.91-0.97)**	**0.95 (0.92-0.99)**	**0.94 (0.91-0.98)**	0.95 (0.89-1.01)
HDL-C	**0.96 (0.92-0.99)**	**0.96 (0.92-0.99)**	0.96 (0.93-1.00)	0.96 (0.92-1.00)	**0.93 (0.87-0.99)**
LDL-C/HDL-C[c]	1.00 (0.96-1.03)	1.00 (0.96-1.03)	1.00 (0.96-1.04)	0.99 (0.95-1.03)	1.00 (0.94-1.07)
TG[c]	0.99 (0.96-1.01)	0.99 (0.96-1.01)	0.98 (0.96-1.01)	0.98 (0.95-1.00)	0.99 (0.95-1.02)
ApoA-I	**0.96 (0.93-0.996)**	**0.96 (0.93-0.996)**	**0.95 (0.91-0.99)**	**0.95 (0.91-0.99)**	**0.92 (0.86-0.99)**
ApoB	0.99 (0.95-1.02)	0.99 (0.95-1.02)	0.99 (0.95-1.03)	0.98 (0.94-1.02)	1.01 (0.94-1.08)
ApoB/ApoA-I[c]	1.00 (0.96-1.04)	1.00 (0.96-1.04)	1.01 (0.96-1.05)	1.01 (0.96-1.06)	1.05 (0.97-1.12)

[a] 563,188 individuals had information on whether they had a history of autoimmune disorders (yes or no) and a history of hospital-treated infections; 492,421 individuals had information on albumin; 407,279 individuals had information on haptoglobin; 344,690 individuals had information on C-reaction protein.

[b] Analyses were adjusted for sex, age at first blood sampling, fasting status at first blood sampling, occupational status, and country of birth.

[c] Logarithmic transformation (log2) was used to the variables of LDL-C/HDL-C ratio, TG, and ApoB/ApoA-I ratio.

TC: total cholesterol; LDL-C: low-density lipoprotein cholesterol; HDL-C: high-density lipoprotein cholesterol; TG: triglycerides; ApoA-I: apolipoprotein A-I; ApoB: apolipoprotein B

Bold font indicates statistical significance.

Table S5. Correlations among the studied metabolic biomarkers

Biomarkers	Glucose	TC	LDL-C	HDL-C	LDL-C/HDL-C[a]	TG[a]	ApoA-I	ApoB	ApoB/ApoA-I[a]
**Glucose**	1	0.131 (0.128,0.133)	0.073 (0.069,0.077)	-0.142 (-0.146,-0.137)	0.150 (0.146,0.154)	0.240 (0.237,0.242)	-0.057 (-0.062,-0.053)	0.1192 (0.114,0.124)	0.126 (0.121,0.131)
**TC**	0.131 (0.128,0.133)	1	0.903 (0.900,0.905)	0.010 (0.005,0.014)	0.554 (0.550,0.557)	0.428 (0.425,0.430)	0.181 (0.1760,0.185)	0.809 (0.806,0.812)	0.601 (0.597,0.605)
**LDL-C**	0.073 (0.069,0.077)	0.903 (0.900,0.905)	1	-0.154 (-0.158,-0.150)	0.692 (0.689,0.695)	0.3137 (0.310,0.318)	-0.080 (-0.084,-0.075)	0.799 (0.796,0.802)	0.721 (0.718,0.724)
**HDL-C**	-0.142 (-0.146,-0.137)	0.010 (0.005,0.014)	-0.154 (-0.158,-0.150)	1	-0.749 (-0.752,-0.747)	-0.519 (-0.522,-0.515)	0.891 (0.889,0.894)	-0.255 (-0.260,-0.251)	-0.633 (-0.637,-0.630)
**LDL-C/HDL-C[a]**	0.150 (0.146,0.154)	0.554 (0.550,0.557)	0.692 (0.689,0.695)	-0.749 (-0.752,-0.747)	1	0.560 (0.556,0.563)	-0.601 (-0.605,-0.597)	0.663 (0.660,0.667)	0.864 (0.861,0.866)
**TG[a]**	0.240 (0.237,0.242)	0.428 (0.425,0.430)	0.3137 (0.310,0.318)	-0.519 (-0.522,-0.515)	0.560 (0.556,0.563)	1	-0.198 (-0.202,-0.194)	0.514 (0.510,0.518)	0.531 (0.527,0.535)
**ApoA-I**	-0.057 (-0.062,-0.053)	0.181 (0.1760,0.185)	-0.080 (-0.084,-0.075)	0.891 (0.889,0.894)	-0.601 (-0.605,-0.597)	-0.198 (-0.202,-0.194)	1	-0.052 (-0.056,-0.047)	-0.520 (-0.524,-0.516)
**ApoB**	0.1192 (0.114,0.124)	0.809 (0.806,0.812)	0.799 (0.796,0.802)	-0.255 (-0.260,-0.251)	0.663 (0.660,0.667)	0.514 (0.510,0.518)	-0.052 (-0.056,-0.047)	1	0.866 (0.864,0.869)
**ApoB/ApoA-I[a]**	0.126 (0.121,0.131)	0.601 (0.597,0.605)	0.721 (0.718,0.724)	-0.633 (-0.637,-0.630)	0.864 (0.861,0.866)	0.531 (0.527,0.535)	-0.520 (-0.524,-0.516)	0.866 (0.864,0.869)	1

[a] Logarithmic transformation (log2) was used to the variables of LDL-C/HDL-C ratio, TG, and ApoB/ApoA-I ratio.

TC: total cholesterol; LDL-C: low-density lipoprotein cholesterol; HDL-C: high-density lipoprotein cholesterol; TG: triglycerides; ApoA-I: apolipoprotein A-I; ApoB: apolipoprotein B.

Table S6. Hazard ratios (HRs) with 95% confidence intervals (CIs) of any hematological malignancy per standard deviation increase of blood biomarkers of lipid, carbohydrate, and apolipoprotein metabolism, analyses further adjusted for other metabolic biomarkers

Biomarker	HR (95% CI)[a]	Further adjusted biomarker(s)
Glucose	0.95 (0.90-1.00)	TG and TC
TC	**0.92 (0.87-0.98)**	TG
LDL-C	**0.85 (0.79-0.92)**	TG
HDL-C	**0.88 (0.81-0.97)**	LDL-C
HDL-C	**0.91 (0.84-0.98)**	TG
LDL-C/HDL-C[b]	**0.91 (0.84-0.99)**	TG
TG[b]	0.92 (0.82-1.04)	LDL-C
TG[b]	0.97 (0.94-1.04)	TC
ApoA-I	**0.90 (0.82-0.99)**	LDL-C
ApoA-I	**0.89 (0.82-0.96)**	TG
ApoB	**0.91 (0.83-0.996)**	TG
ApoB/ApoA-I[b]	0.91 (0.83-1.00)	TG

[a] Analyses were adjusted for sex, age at first blood sampling, fasting status at first blood sampling, occupational status, and country of birth.

[b] Logarithmic transformation (log2) was used to the variables of LDL-C/HDL-C ratio, TG, and ApoB/ApoA-I ratio.

TC: total cholesterol; LDL-C: low-density lipoprotein cholesterol; HDL-C: high-density lipoprotein cholesterol; TG: triglycerides; ApoA-I: apolipoprotein A-I; ApoB: apolipoprotein B

Bold font indicates statistical significance.

Table S7. Hazard ratios (HRs) with 95% confidence intervals (CIs) of hematological malignancy per standard deviation increase of blood biomarkers of lipid, carbohydrate, and apolipoprotein metabolism, analyses restricted to first blood collection in relation to an occupational health check-up

**Biomarker**	**Any hematological malignancy**	**Acute myeloid leukemia**	**Multiple myeloma**	**Hodgkin's lymphoma**	**Non-Hodgkin's lymphoma**	**Chronic lymphoid leukemia**
	**HR (95% CI)[a]**	**HR (95% CI)[a]**	**HR (95% CI)[a]**	**HR (95% CI)[a]**	**HR (95% CI)[a]**	**HR (95% CI)[a]**
Glucose	1.00 (0.97-1.03)	1.01 (0.91-1.12)	1.01 (0.95-1.07)	1.14 (1.02-1.28)	0.96 (0.92-1.01)	1.06 (1.00-1.12)
TC	**0.94 (0.91-0.96)**	0.94 (0.86-1.04)	**0.89 (0.83-0.94)**	0.99 (0.84-1.17)	**0.96 (0.92-0.995)**	0.97 (0.91-1.04)
LDL-C	**0.93 (0.90-0.97)**	0.95 (0.82-1.09)	0.89 (0.82-0.97)	1.04 (0.81-1.33)	**0.94 (0.89-0.99)**	0.99 (0.90-1.09)
HDL-C	0.96 (0.93-1.00)	0.83 (0.73-1.96)	0.97 (0.89-1.06)	1.09 (0.85-1.39)	0.96 (0.91-1.02)	0.97 (0.88-1.07)
LDL-C/HDL-C[b]	0.99 (0.95-1.03)	1.08 (1.02-1.15)	0.95 (0.87-1.03)	1.00 (0.78-1.28)	1.00 (0.94-1.05)	1.02 (0.93-1.12)
TG[b]	0.98 (0.96-1.01)	0.99 (0.91-1.09)	0.99 (0.94-1.05)	1.08 (0.92-1.27)	0.96 (0.93-1.00)	1.02 (0.95-1.08)
ApoA-I	0.97 (0.93-1.01)	0.86 (0.74-1.00)	0.96 (0.88-1.06)	1.06 (0.81-1.38)	0.95 (0.89-1.01)	1.04 (0.94-1.15)
ApoB	0.97 (0.93-1.01)	0.98 (0.84-1.15)	0.93 (0.84-1.03)	1.11 (0.84-1.47)	1.01 (0.95-1.07)	0.99 (0.89-1.10)
ApoB/ApoA-I[b]	0.99 (0.94-1.03)	1.06 (0.90-1.25)	0.97 (0.88-1.08)	1.15 (0.86-1.55)	1.03 (0.96-1.10)	0.96 (0.86-1.07)

[a] Analyses were adjusted for sex, age at first blood sampling, fasting status at first blood sampling, occupational status, and country of birth.

[b] Logarithmic transformation (log2) was used to the variables of LDL-C/HDL-C ratio, TG, and ApoB/ApoA-I ratio.

TC: total cholesterol; LDL-C: low-density lipoprotein cholesterol; HDL-C: high-density lipoprotein cholesterol; TG: triglycerides; ApoA-I: apolipoprotein A-I; ApoB: apolipoprotein B

Bold font indicates statistical significance.

Table S8. Hazard ratios (HRs) with 95% confidence intervals (CIs) of hematological malignancy per standard deviation increase of blood biomarkers of lipid, carbohydrate, and apolipoprotein metabolism, analyses adjusted for multiple comparison using Bonferroni correction.

Biomarker	Any hematological malignancy	Acute myeloid leukemia	Multiple myeloma	Hodgkin's lymphoma	Non-Hodgkin's lymphoma	Chronic lymphoid leukemia
	HR (95% CI)[a]	HR (95% CI)[a]	HR (95% CI)[a]	HR (95% CI)[a]	HR (95% CI)[a]	HR (95% CI)[a]
Glucose	1.00 (0.97-1.03)	0.97 (0.82-1.12)	1.02 (0.94-1.10)	1.13 (0.93-1.33)	0.96 (0.90-1.02)	1.04 (0.95-1.13)
TC	**0.93 (0.89-0.97)**	0.92 (0.79-1.05)	**0.89 (0.81-0.97)**	0.97 (0.71-1.23)	0.95 (0.90-1.00)	0.98 (0.89-1.07)
LDL-C	**0.94 (0.89-0.99)**	0.91 (0.72-1.10)	0.90 (0.77-1.03)	0.95 (0.57-1.33)	0.96 (0.88-1.04)	1.00 (0.85-1.15)
HDL-C	0.96 (0.90-1.02)	0.86 (0.67-1.05)	0.96 (0.83-1.09)	1.10 (0.66-1.54)	0.95 (0.87-1.03)	0.96 (0.82-1.10)
LDL-C/HDL-C[b]	1.00 (0.94-1.06)	1.07 (0.93-1.21)	0.96 (0.82-1.10)	0.93 (0.56-1.30)	1.01 (0.93-1.09)	1.03 (0.89-1.17)
TG[b]	0.99 (0.95-1.03)	0.99 (0.85-1.13)	1.00 (0.92-1.08)	1.07 (0.80-1.34)	0.96 (0.90-1.02)	1.02 (0.92-1.12)
ApoA-I	0.96 (0.90-1.02)	0.88 (0.68-1.08)	0.96 (0.82-1.10)	1.06 (0.61-1.51)	0.93 (0.84-1.02)	1.01 (0.86-1.16)
ApoB	0.99 (0.93-1.05)	0.97 (0.74-1.20)	0.97 (0.83-1.11)	1.06 (0.59-1.53)	1.01 (0.91-1.11)	1.01 (0.85-1.17)
ApoB/ApoA-I[b]	0.98 (0.92-1.04)	1.02 (0.77-1.27)	1.00 (0.84-1.16)	1.13 (0.60-1.66)	1.04 (0.94-1.14)	0.99 (0.82-1.16)

[a] Analyses were adjusted for sex, age at first blood sampling, fasting status at first blood sampling, occupational status, and country of birth.

[b] Logarithmic transformation (log2) was used to the variables of LDL-C/HDL-C ratio, TG, and ApoB/ApoA-I ratio.

TC: total cholesterol; LDL-C: low-density lipoprotein cholesterol; HDL-C: high-density lipoprotein cholesterol; TG: triglycerides; ApoA-I: apolipoprotein A-I; ApoB: apolipoprotein B

Bold font indicates statistical significance.

Table S9. Hazard ratios (HRs) with 95% confidence intervals (CIs) of hematological malignancy in relation to different levels of blood biomarkers of lipid, carbohydrate, and apolipoprotein metabolism, analyses using the clinical cutoff values of the biomarkers

Biomarker	Any hematological malignancy	Acute myeloid leukemia	Myeloma	Hodgkin's lymphoma	Non-Hodgkin's lymphoma	Chronic lymphoid leukemia
	HR (95% CI)[a]	HR (95% CI)[a]	HR (95% CI)[a]	HR (95% CI)[a]	HR (95% CI)[a]	HR (95% CI)[a]
**Glucose (mmol/l)**						
<3.90	1.12 (0.95-1.34)	0.79 (0.38-1.68)	1.26 (0.84-1.87)	1.03 (0.32-3.27)	1.25 (0.97-1.61)	0.92 (0.54-1.56)
≥3.90 and <5.60	1.0 (ref)	1.0 (ref)	1.0 (ref)	1.0 (ref)	1.0 (ref)	1.0 (ref)
≤5.60	**0.92 (0.85-0.997)**	0.84 (0.62-1.15)	1.01 (0.84-1.21)	0.96 (0.52-1.78)	0.82 (0.72-0.93)	1.02 (0.83-1.25)
**TC (mmol/l)**						
<6.50	1.0 (ref)	1.0 (ref)	1.0 (ref)	1.0 (ref)	1.0 (ref)	1.0 (ref)
≥6.50	**0.94 (0.88-0.996)**	0.95 (0.75-1.19)	0.93 (0.80-1.08)	0.72 (0.43-1.21)	0.92 (0.84-1.01)	1.10 (0.88-1.37)
**LDL-C (mmol/l)**						
<4.10	1.0 (ref)	1.0 (ref)	1.0 (ref)	1.0 (ref)	1.0 (ref)	1.0 (ref)
≥4.10	0.94 (0.87-1.02)	0.94 (0.70-1.28)	0.85 (0.70-1.03)	1.07 (0.61-1.89)	1.01 (0.88-1.14)	0.90 (0.64-1.27)
**HDL-C (mmol/l)**						
<1.03	1.14 (1.01-1.28)	1.29 (0.84-1.97)	1.08 (0.82-1.44)	0.88 (0.37-2.08)	1.14 (0.95-1.37)	1.19 (0.93-1.53)
≥1.03	1.0 (ref)	1.0 (ref)	1.0 (ref)	1.0 (ref)	1.0 (ref)	1.0 (ref)
**LDL-C/HDL-C**						
<3.50	1.0 (ref)	1.0 (ref)	1.0 (ref)	1.0 (ref)	1.0 (ref)	1.0 (ref)
≥3.50	1.00 (0.91-1.10)	0.85 (0.59-1.23)	1.01 (0.81-1.26)	1.00 (0.52-1.93)	0.97 (0.83-1.13)	1.13 (0.95-1.35)
**TG (mmol/l)**						
<1.71	1.0 (ref)	1.0 (ref)	1.0 (ref)	1.0 (ref)	1.0 (ref)	1.0 (ref)
≥1.71	1.05 (0.98-1.12)	1.04 (0.85-1.31)	1.11 (0.95-1.30)	**1.63 (1.03-2.58)**	0.93 (0.86-1.01)	**1.14 (1.003-1.29)**

**ApoA-I (g/l)**						
<1.05	0.91 (0.72-1.16)	1.77 (0.93-3.37)	0.85 (0.48-1.51)	1.06 (0.26-4.37)	0.97 (0.87-1.08)	0.95 (0.51-1.79)
≥1.05	1.0 (ref)	1.0 (ref)	1.0 (ref)	1.0 (ref)	1.0 (ref)	1.0 (ref)
**ApoB (g/l)**						
<1.50	1.0 (ref)	1.0 (ref)	1.0 (ref)	1.0 (ref)	1.0 (ref)	1.0 (ref)
≥1.50	1.07 (0.97-1.18)	1.12 (0.79-1.57)	1.12 (0.90-1.40)	1.29 (0.65-2.57)	0.92 (0.64-1.34)	1.14 (0.88-1.46)
**ApoB/ApoA-I**						
<1	1.0 (ref)	1.0 (ref)	1.0 (ref)	1.0 (ref)	1.0 (ref)	1.0 (ref)
≥1	1.06 (0.97-1.16)	1.14 (0.82-1.58)	1.03 (0.83-1.27)	1.37 (0.62-2.60)	1.15 (1.00-1.12)	1.04 (0.81-1.32)

[a] Analyses were adjusted for sex, age at first blood sampling, fasting status at first blood sampling, occupational status, and country of birth.

TC: total cholesterol; LDL-C: low-density lipoprotein cholesterol; HDL-C: high-density lipoprotein cholesterol; TG: triglycerides; ApoA-I: apolipoprotein A-I; ApoB: apolipoprotein B

Bold font indicates statistical significance.

Table S10. Hazard ratios (HRs) with 95% confidence intervals (CIs) of any hematological malignancy per standard deviation increase of blood biomarkers of lipid, carbohydrate, and apolipoprotein metabolism, analyses further adjusted for diagnosis of diabetes, BMI, or smoking[a]

Biomarker	Further adjustment for diagnosis of diabetes	Further adjustment for BMI	Further adjustment for smoking
	**HR (95% CI)[b]**	**HR (95% CI)[b]**	**HR (95% CI)[b]**
Glucose	1.00 (0.98-1.02)	1.01 (0.95-1.08)	1.07 (0.86-1.33)
TC	**0.93 (0.91-0.96)**	**0.93 (0.88-0.99)**	0.86 (0.71-1.04)
LDL-C	**0.94 (0.90-0.97)**	0.99 (0.91-1.08)	0.99 (0.91-1.08)
HDL-C	**0.96 (0.92-0.99)**	0.91 (0.83-1.00)	0.91 (0.83-1.00)
LDL-C/HDL-C[c]	0.99 (0.96-1.03)	1.05 (0.96-1.15)	1.05 (0.96-1.15)
TG[c]	0.99 (0.96-1.01)	1.01 (0.95-1.07)	1.01 (0.85-1.20)
ApoA-I	**0.96 (0.93-0.996)**	0.96 (0.88-1.05)	0.96 (0.88-1.05)
ApoB	0.98 (0.95-1.02)	1.07 (0.98-1.17)	1.02 (0.71-1.48)
ApoB/ApoA-I[c]	1.00 (0.96-1.04)	1.08 (0.98-1.18)	1.08 (0.98-1.18)

[a] 563,188 individuals had information on whether they had a diagnosis of diabetes (yes or no); 104,936 individuals had information on BMI; 38,648 individuals had information on smoking.

[b] Analyses were adjusted for sex, age at first blood sampling, fasting status at first blood sampling, occupational status, and country of birth.

[c] Logarithmic transformation (log2) was used to the variables of LDL-C/HDL-C ratio, TG, and ApoB/ApoA-I ratio.

TC: total cholesterol; LDL-C: low-density lipoprotein cholesterol; HDL-C: high-density lipoprotein cholesterol; TG: triglycerides; ApoA-I: apolipoprotein A-I; ApoB: apolipoprotein B

Bold font indicates statistical significance.
